# Surface Plasmon Resonance Imaging Sensor for Detection of Photolytically and Photocatalytically Degraded Glyphosate

**DOI:** 10.3390/s22239217

**Published:** 2022-11-27

**Authors:** Martina Vráblová, Kateřina Smutná, Ivan Koutník, Tomáš Prostějovský, Radim Žebrák

**Affiliations:** 1 Institute of Environmental Technology, CEET, VSB-Technical University of Ostrava, 17. listopadu 15, 708 00 Ostrava, Czech Republic; 2 Faculty of Materials Science and Technology, VSB-Technical University of Ostrava, 17. listopadu 15, 708 00 Ostrava, Czech Republic; 3 Dekonta Inc., Dřetovice 109, 273 42 Stehelčeves, Czech Republic

**Keywords:** glyphosate, pesticide, photocatalysis, surface plasmon resonance, aminomethylphosphonic acid

## Abstract

Glyphosate is one of the most widely used pesticides, which, together with its primary metabolite aminomethylphosphonic acid, remains present in the environment. Many technologies have been developed to reduce glyphosate amounts in water. Among them, heterogeneous photocatalysis with titanium dioxide as a commonly used photocatalyst achieves high removal efficiency. Nevertheless, glyphosate is often converted to organic intermediates during its degradation. The detection of degraded glyphosate and emerging products is, therefore, an important element of research in terms of disposal methods. Attention is being paid to new sensors enabling the fast detection of glyphosate and its degradation products, which would allow the monitoring of its removal process in real time. The surface plasmon resonance imaging (SPRi) method is a promising technique for sensing emerging pollutants in water. The aim of this work was to design, create, and test an SPRi biosensor suitable for the detection of glyphosate during photolytic and photocatalytic experiments focused on its degradation. Cytochrome P450 and TiO_2_ were selected as the detection molecules. We developed a sensor for the detection of the target molecules with a low molecular weight for monitoring the process of glyphosate degradation, which could be applied in a flow-through arrangement and thus detect changes taking place in real-time. We believe that SPRi sensing could be widely used in the study of xenobiotic removal from surface water or wastewater.

## 1. Introduction

The use of pesticides in modern agriculture brings benefits as well as problems. The environmental risk of using pesticides is their persistence in soil, water, and air, either in their original form or in the form of their transformation products. One of the most heavily applied pesticides has been glyphosate (*N*-(phosphonomethyl)glycine), which is a broad-spectrum organophosphate herbicide used for weed control. It was brought to market under the trade name Roundup by the Monsanto Company in the 1970s. Genetically modified “roundup ready” (glyphosate-tolerant) crops were introduced in the 1990s, which further supported the expansion of glyphosate use. Glyphosate and its primary metabolite aminomethylphosphonic acid (AMPA) remain present not only in the environment but their residues are found in crops and processed food as well [1,2]. In 2015, glyphosate was classified as “probably carcinogenic to humans” by the WHO’s International Agency for Research on Cancer [3]. Glyphosate exposure has been linked to an increased risk of non-Hodgkin’s lymphoma, endocrine disruptions, and chromosomal damage [4,5]. Feulefack et al. [6] confirmed an association between prenatal exposure to organophosphate herbicides and childhood brain tumors. Although glyphosate application has been banned or regulated in many countries, its usage and human exposure to glyphosate-based herbicides continue to rise. Due to adverse health outcomes and expected carcinogenicity risk, it is desirable to deplete the content of glyphosate in the environment.

Glyphosate concentration is usually determined by chromatographic methods (gas chromatography and liquid chromatography). Among them, liquid chromatography–tandem mass spectrometry (LC–MS/MS) is the most commonly used for its accuracy and low limit of detection [7]. Chromatographic methods are also suitable for AMPA detection [8,9]. However, chromatography is costly and time-consuming. Other possibilities for glyphosate determination are UV–vis and Raman spectroscopy [10,11,12], nuclear magnetic resonance [13], and enzyme-linked immunosorbent assay (ELISA) [14,15]. Capillary electrophoresis enables the determination of not only glyphosate but also its metabolites glufosinate and AMPA [16]. A promising method for the online detection of organic compounds is surface plasmon resonance (SPR) [17,18,19]. It is an optical method used for a label-free real-time interaction analysis between a ligand, which is bound on a gold surface of a biochip, and an analyte flowing over the biochip surface. During SPRi measurement, the area of the biochip is scanned; therefore, more than one ligand can be bound on the biochip surface, and multi-analyte analysis is possible in real time. Ding and Yang [20] reported an SPR biosensor with an oligopeptide as a sensing element for the online detection of glyphosate. This SPR biosensor also showed good specificity against other analytes such as glycine, thiacloprid, and imidacloprid. Do et al. [21] described the association of SPR with chitosan (CS) film and its nanocomposites, including zinc oxide (ZnO) or graphene oxide for glyphosate detection. The sorption of both glyphosate and its degradation product AMPA on the CS/ZnO SPR sensor was observed.

Many technologies have been developed to reduce glyphosate amounts in water and soil, including physical, chemical, and biological methods [22]. The cheapest and most eco-friendly method of removing glyphosate from the environment is bioremediation [23]. During microbial degradation, soil bacteria utilize glyphosate as the sole phosphorus, carbon, or nitrogen source. Three main glyphosate metabolites, AMPA, sarcosine, and acetylglyphosate, are further metabolized through various pathways [24]. The complete biodegradation of glyphosate and its metabolites results in inorganic phosphate, ammonium, and carbon dioxide formation [25]. However, the disadvantage of biological methods for the degradation of glyphosate is, in particular, a long residence time compared to nonbiological technologies.

The available nonbiological technologies for glyphosate removal include membrane separation [26,27], adsorption [28], advanced oxidation processes (AOPs), photocatalysis, and their combinations. Although adsorption itself does not degrade glyphosate molecules, it can deplete glyphosate concentration in water significantly [29]. Advanced oxidation processes (AOPs) exhibit a high glyphosate removal efficiency. The simple combination of hydrogen peroxide and UV radiation (H_2_O_2_/UV) represents a low-cost technology for glyphosate degradation with more than 70% conversion [30]. Complete glyphosate removal has been obtained by Fenton-based processes, electrochemical oxidation, and photocatalysis [31,32,33]. A commonly used heterogeneous photocatalyst used for glyphosate removal has been titanium dioxide. The photocatalytic degradation involves adsorption on the TiO_2_ surface and photocatalysis, during which the organic intermediates sarcosine and AMPA are mineralized to phosphates, nitrates, and formic acid and are subsequently decarboxylated to carbon dioxide [32].

This work aimed to design, create, and test an SPRi biosensor suitable for the detection of glyphosate during photolytic and photocatalytic experiments focused on its degradation. Cytochrome P450 and TiO_2_ were selected as the detection molecules; the design of a biochip suitable for the detection of glyphosate was based on the known glyphosate inhibition of cytochrome P450 enzymes [34] and the known photocatalytic activity of TiO_2_ (Evonik P25) for glyphosate degradation [32], which presupposes their mutual interactions. Another goal was to monitor the effect of increasing the concentration of AMPA on glyphosate detection during the experiments. The photolytic and photocatalytic tests were performed in a homemade batch photoreactor with or without the commercial photocatalyst TiO_2_ P25, respectively.

## 2. Material and Methods

### 2.1. Chemicals

**Scheme 1:**
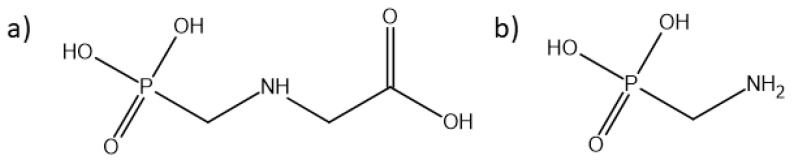
Molecular structure of (**a**) glyphosate and (**b**) its degradation product aminomethylphosphonic acid (AMPA).

Photolytic and photocatalytic degradation experiments were performed with glyphosate ((*N*-(phosphonomethyl)glycine, 96%, Sigma-Aldrich, Steinheim, Germany). Analytical methods were calibrated using glyphosate and aminomethylphosphonic acid (AMPA) analytical standards (HPC Standards GmbH, Cunnersdorf, Germany) (Scheme 1). Cytochrome P450 human 1A2 (Sigma-Aldrich, Steinheim, Germany), supplied by the manufacturer as a solution containing ≥ 0.01 mg L^−1^ of protein, was used for SPRi detection. TiO_2_ P25 (Sigma-Aldrich, Steinheim, Germany) was used in photocatalytic degradation experiments and for SPRi detection.

### 2.2. Surface Plasmon Resonance Imaging (SPRi) and a Biochip Preparation

A bare SPRi-Biochip™ with a gold surface (HORIBA France SAS, Longjumeau, France) was spotted with an aqueous solution of cytochrome and a suspension of TiO_2_. Cytochrome was diluted ten times in ultrapure water (18.2 MΩ cm) to a final concentration of protein ≥1 g L^−1^. A suspension (1 wt.%) of TiO_2_ in ethanol (96% p.a., Lach-Ner, Neratovice, Czech Republic) was prepared. Then, the suspension was diluted in ultrapure water to 0.01 wt.%. Drops of approximately 1 μL of each solution were applied to the gold surface of the biochip and left overnight in a dark and humid atmosphere to immobilize. Then, the biochip was washed with ultrapure water and air-dried. The process of the preparation of ligand (cytochrome and TiO_2_) solutions and their immobilization on a biochip surface has been tested several times and we presented only the most suitable one.

**Figure 1:**
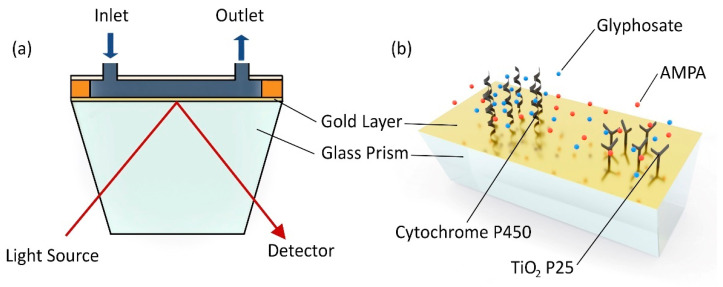
(**a**) A scheme of the SPRi principle and (**b**) a biochip surface with immobilized cytochrome P450 and TiO_2_ P25.

A scheme of an SPRi instrument and a biochip arrangement is shown in Figure 1. The measurement was performed on an OpenPlex SPRi instrument (HORIBA France SAS, Longjumeau, France). Optical excitation of surface plasmons was achieved by the method of attenuated total reflection (prism coupling). The measurements were performed at a fixed angle, and the amplitude (reflectivity in %) was measured. Measuring spots corresponding to cytochrome and TiO_2_ were selected by software at the beginning of the measurement. The use of the imaging mode made it possible to measure the concentration of analyte on both the cytochrome and TiO_2_ spots at the same time. The mobile phase (ultrapure water) was degassed through a vacuum degasser and pumped into the apparatus with a constant flow (50 μL min^−1^) via a peristaltic pump. The measurement of the prepared samples was performed by injecting the sample with the analyte(s) through the flow loop (volume 200 μL). Samples were prepared by taking 1 mL of solution from the photoreactor and filtered through a syringe filter with regenerated cellulose (pore size 0.22 μm). The biochip was calibrated for glyphosate, AMPA, and a mixture of them dissolved in ultrapure water in various concentrations in the range from 10–100 mg L^−1^ during 8 measuring days. The detection limit for glyphosate was 3.7 mg L^−1^. Calibration for glyphosate was performed before each measurement of real samples from photolytic and photocatalytic degradation experiments. Between measurements, the biochip was placed in the instrument under a flow of ultrapure water.

### 2.3. Total Organic Carbon (TOC) Analysis

Samples for TOC analysis were prepared by taking 3 mL samples from the photoreactor and then diluting them with 3 mL of distilled water. Samples with photocatalyst were first filtered through a syringe filter with regenerated cellulose (pore size 0.22 μm). All samples from photolytic and photocatalytic degradation experiments were analyzed on a total carbon analyzer (Formacs^HT-I^, Skalar Ltd., Breda, The Netherlands). The quantity of non-purgeable organic carbon (NPOC) was determined.

### 2.4. Liquid Chromatography-Mass Spectrometry (LC–MS) Analysis

LC–MS analyses were carried out on Shimadzu Nexera XR series HPLC instrument (Shimadzu, Japan) coupled to a QTRAP 6500+ mass spectrometer (Sciex, Framingham, MA, USA) equipped with an electrospray ionization source. A Restek Raptor Polar X column (50 × 2.1 mm i.d.) was used (flow rate 0.5 mL min^−1^). The mobile phases were 0.5% formic acid in water (A) and 0.5% formic acid in acetonitrile (B) in the gradient mode. MS parameters (ESI-) were −4500 V and 500 °C. Multiple reaction monitoring (MRM) was used for the measuring of glyphosate and AMPA; glyphosate: MRM1 167.887 → 149.8, MRM2 167.887 → 62.8; AMPA: MRM1 110 → 78.9; MRM2 110 → 62.9.

### 2.5. Photolytic and Photocatalytic Degradation of Glyphosate

Photolytic and photocatalytic experiments were carried out in a stainless-steel batch photoreactor (volume 305 mL). Glyphosate solution had a concentration 100 mg L^−1^ and volume 100 mL. An 8W UV pen-ray lamp (*λ*_peak_ = 254 nm, Ultra-Violet Products Inc., Cambridge, UK) was used as an irradiation source and was laid on top of a quartz glass window on top of the photoreactor. The solution was irradiated for 4 h and liquid samples were taken at 0, 1, 2, 3, and 4 h for TOC, HPLC, and SPRi analyses.

In the case of photocatalysis, the experiments were conducted with a powder commercial photocatalyst TiO_2_ P25 (Sigma-Aldrich, Steinheim, Germany). In the dark, 0.1 g of the photocatalyst was added into 100 mL of the glyphosate solution (photocatalyst concentration 1 g L^−1^), stirred for 30 min to reach adsorption–desorption equilibria, and then a liquid sample was taken and analyzed. Then, the lamp was turned on and the photocatalytic reaction started. Liquid samples for TOC, HPLC, and SPRi analyses were taken at the same time intervals as in the case of photolysis.

Data acquired from HPLC analysis were used to calculate rate constants for the photolytic and photocatalytic reduction of glyphosate (Equations (1) and (2)).

(1) $$\upsilon =\frac{dc}{d\tau }=k_{app}\cdot c$$

(2) $$ln\frac{c_{0}}{c}=k_{app}\cdot \tau$$

where υ is the glyphosate reduction rate (mg L^−1^ min^−1^), c is the glyphosate concentration (mg L^−1^), τ is the irradiation time (min), $k_{app}$ is the apparent glyphosate reduction constant (min^−1^), and $c_{0}$ is the initial glyphosate concentration (mg L^−1^). The degradation followed a pseudo-first-order kinetics thus the rate constants were only apparent.

### 2.6. Data Processing

Data were processed and plotted using the OriginPro software (2018b, OriginLab Corporation, Northampton, MA, USA) and the Spectragryph optical spectroscopy software (F. Menges “Spectragryph—optical spectroscopy software”, Version 1.2.15, 2020).

## 3. Results and Discussion

In our study, the photolytic and photocatalytic degradation of glyphosate was detected by the three independent methods of surface plasmon resonance imaging (SPRi), total organic carbon analysis (TOC), and liquid chromatography coupled with mass spectrometry (HPLC–MS).

The principle of SPR biosensors presupposes a specific interaction of the monitored analyte with the surface of the biochip [35]. It was, therefore, necessary to find (bio)molecules that could be immobilized on the surface of the biochip and that would interact with glyphosate while being sufficiently stable to allow the surface to be used for analysis over a longer time. Previously, chitosan-based nanocomposites [21] or oligopeptides [20] were used as detection molecules for glyphosate. There are also valuable works that do not use SPR detection but publish interactions between compounds, as in the case of Samsel and Seneff [34], who described glyphosate’s suppression of cytochrome P450 enzymes and amino acid biosynthesis by the gut microbiome. On the other hand, inorganic compounds or nanoparticles are more stable than biomolecules and can also be used in the detection of organic substances, e.g., in the study by Zhang et al. [36], who used titanium dioxide nanoparticles for the modification of plasmonic interface for biomolecular sensing. In this work, we combined cytochrome P450 and TiO_2_ to modify the golden biochip surface for glyphosate sensing.

We tested the sensitivity of both the cytochrome and TiO_2_ spots for glyphosate and its degradation product AMPA. The calibration curves of glyphosate and AMPA revealed a linear relationship between an SPR signal (reflectivity) and the concentration on both the cytochrome and TiO_2_ spots (Figure S1), with the regression coefficients of determination of adj. R-square > 98. The measurements were performed within several days when good repeatability and time stability of the biochip were proved by the high value of the adj. R-square, when the data measured on different days were pooled. The use of ultrapure water as the mobile phase instead of a buffer, which is commonly used when proteins are selected as ligands in SPR analysis [17,37], was not an obstacle either.

The detection of glyphosate was more sensitive on cytochrome than TiO_2_, with slopes of linear regression of 0.0052 and 0.0017, respectively. For AMPA, the sensitivity was the same on cytochrome and TiO_2_ (0.0014 vs. 0.0013). The higher response on the cytochrome spot for glyphosate suggested a specific interaction between the two molecules. This was not surprising, as cytochrome P450 is found in liver cells, where it metabolizes drugs and other foreign substances, and a good affinity between an enzyme and a broad spectrum of metabolized compounds is, therefore, necessary [38].

The detection of two structurally similar compounds on one ligand (a molecule immobilized on the biochip surface) can be affected by their interference. To solve this problem, it is necessary to use two or more ligands, on which the detection will take place, with different sensitivity, and one of them can be used as a negative control [39]. The different detection sensitivity for glyphosate and AMPA between the spots of cytochrome and TiO_2_ led us to monitor the cytochrome/TiO_2_ signal ratio in the model solutions containing a mixture of glyphosate and AMPA (Figure S2). The total concentration of substances in the solution was 100 mg L^−1^ and the ratio of glyphosate to AMPA varied. The relationship between the SPR signal (reflectivity) and the concentration of glyphosate in the mixture was linear (adj. R-square 0.93).

**Figure 2:**
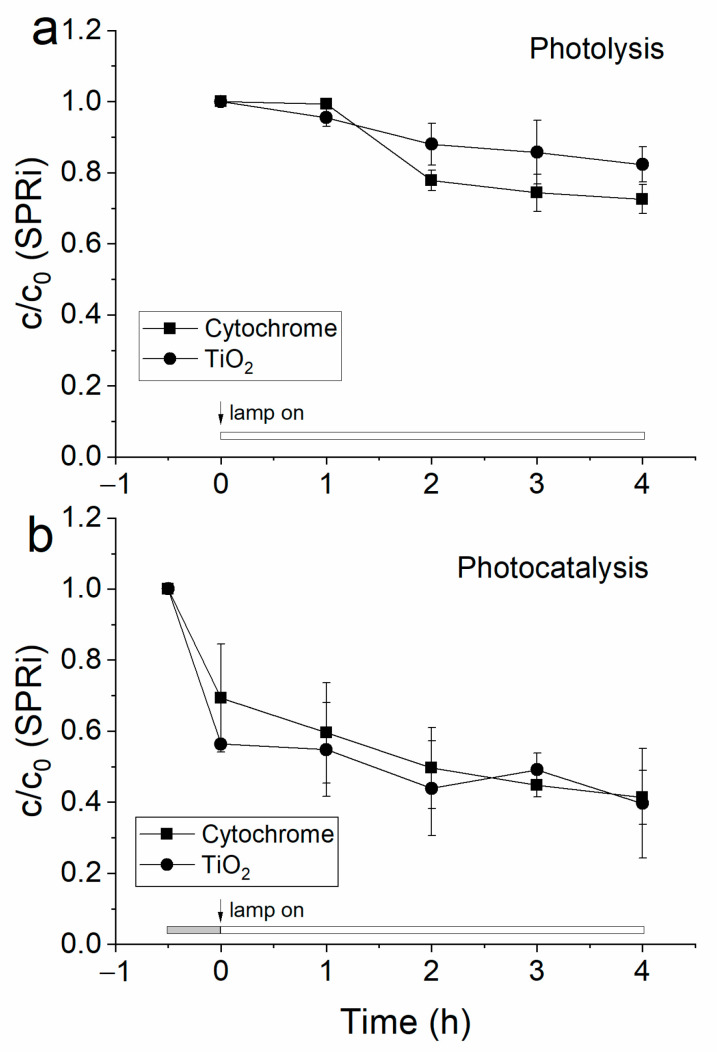
SPRi analysis of a glyphosate solution with expected by-products formed during (**a**) photolysis and (**b**) photocatalysis experiments. The initial concentration of glyphosate was 100 mg L^−1^. Each measurement was repeated three times. The mean values and the standard errors of the determination are shown.

Surface plasmon resonance imaging revealed a decrease in the signal measured on both the cytochrome and TiO_2_ spots over the observed period in both types of degradation experiments (Figure 2). A decrease in signal appeared during the dark phase of photocatalysis (Figure 2b) when the adsorption of glyphosate onto the TiO_2_ particles took place. The drop in the reflectivity over time was similar on both cytochrome and TiO_2_, with a maximum reduction in the signal of 28% and 60% in photolysis and photocatalysis, respectively.

**Figure 3:**
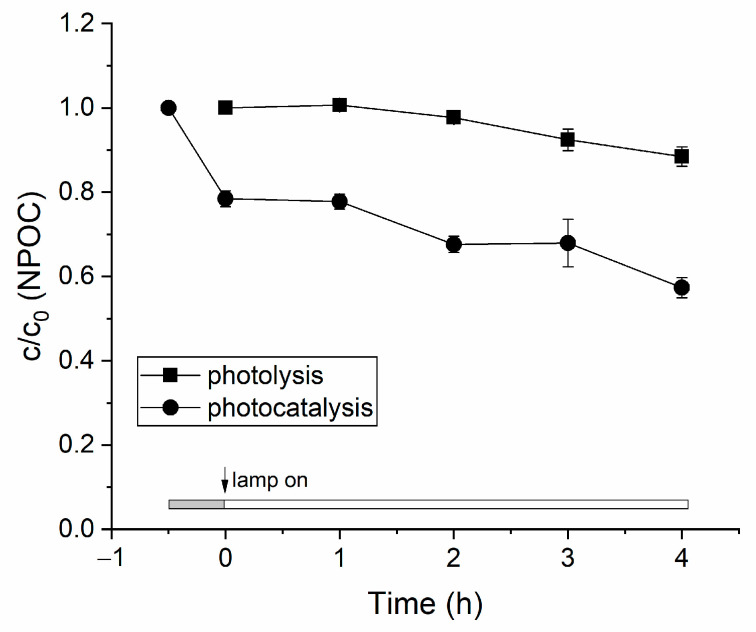
Analysis of total organic carbon during photolytic and photocatalytic degradation of glyphosate. The initial concentration of glyphosate was 100 mg L^−1^. Each measurement was repeated three times. The mean values and the standard errors of the determination are shown.

TOC analysis revealed a decrease in the concentration of organic compounds (NPOC) in the photoreactor over time (Figure 3). Photolysis reduced the organic content by 11.5% after 4 h, while in the presence of the photocatalyst there was a reduction of 43.0%, of which 21.3% was due to adsorption of glyphosate on the photocatalyst and the rest was due to photocatalytic decomposition during 4 h of irradiation. Within the first hour of exposure to UV rays, the lag phase appeared with a plateau in the carbon content in both photolysis and photocatalysis.

**Figure 4:**
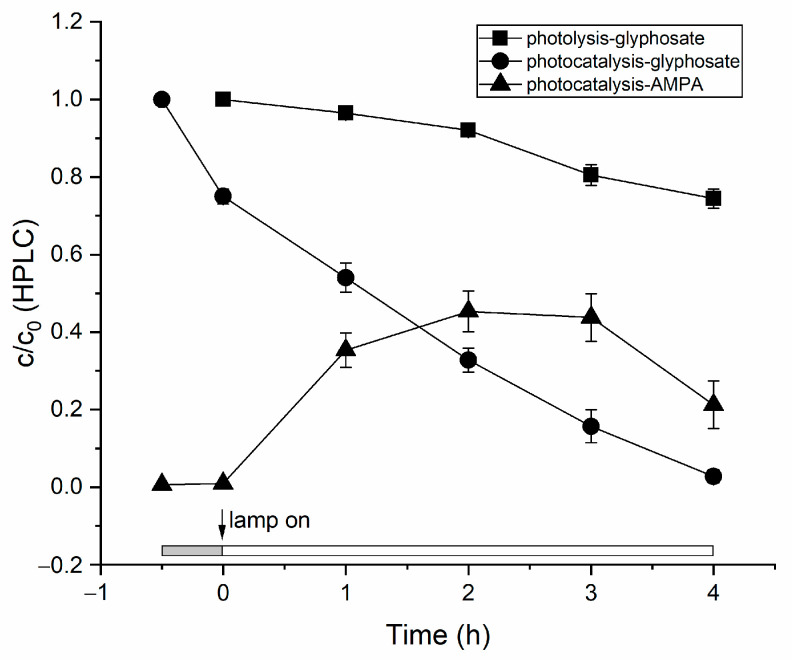
HPLC–MS analysis of glyphosate and AMPA during photolytic and photocatalytic degradation experiments. The initial concentration of glyphosate was 100 mg L^−1^. Each measurement was repeated three times. The mean values and the standard errors of the determination are shown.

The observed lower concentration of compounds in the samples measured on SPRi compared to TOC was because TOC also measures very small molecules, to which the biosensor surface may no longer be sensitive. A mixture of substances (glyphosate and its degradation products) is detected in both TOC and SPRi analyses. Therefore, it is appropriate to include a targeted analytical method to verify the decrease in glyphosate concentration during its degradation when introducing a new sensory method. In our case, the HPLC–MS method was used for the identification and quantification of glyphosate and AMPA, which was expected to occur during experiments [32,40]. Similar to the TOC and SPRi measurements, the glyphosate concentration decreased over time in both the photolytic and photocatalytic degradation experiments (Figure 4).

The photolytic reduction of glyphosate measured by liquid chromatography was 26%, whereas the reduction by photocatalysis achieved almost 100%. The calculated rate constants for the photolytic and photocatalytic reduction of glyphosate were 1.13 × 10^−3^ min^−1^ and 1.20 × 10^−2^ min^−1^, respectively. AMPA was identified as the major degradation product of glyphosate. Its concentration increased to a maximum of 45 mg L^−1^ after 2–3 h from the beginning of UV-light exposure, which, at the original glyphosate concentration after the dark phase of 75 mg L^−1^, meant a 60% conversion of the glyphosate to AMPA. In the last hour of the experiment, the concentration of AMPA decreased, which meant that there was probably further degradation to another carbonaceous compound. In the photolysis experiments, AMPA was identified in the samples, but the quantification failed due to the presence of other unidentified product(s) with similar molecular structures. Thus, compared to the SPR and TOC analyses, HPLC yielded more accurate results for the final glyphosate concentration, as it was free from interference from the degradation product formed during the photolysis and photocatalysis. However, if targeted HPLC analysis is used, other degradation products may not be uncovered.

**Figure 5:**
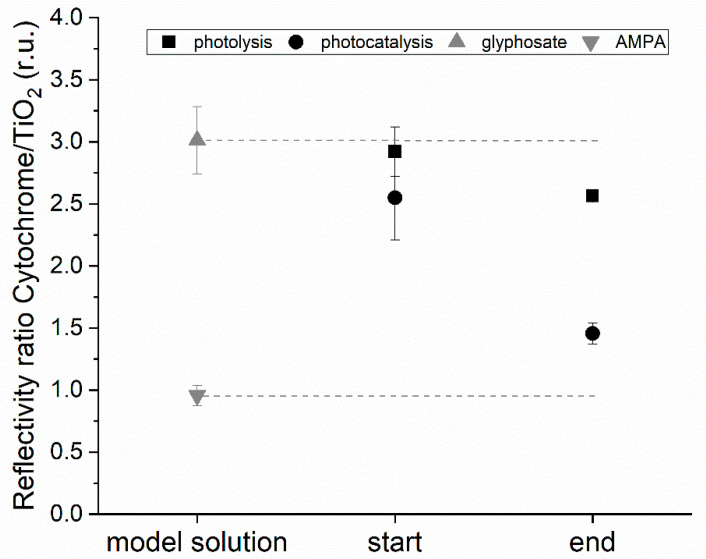
Signal ratio obtained on cytochrome and TiO_2_ for standard solutions of glyphosate and AMPA and for samples from the beginning (start) and the end (end) of photolytic and photocatalytic degradation experiments. For standard solutions, n = 16 (glyphosate) and 8 (AMPA). Photolytic and photocatalytic degradation measurements were repeated three times. The mean values and the standard errors of the determination are shown.

It was shown that the SPRi analysis allowed the fast and simple detection of glyphosate dissolved in water (Figures S1 and S2). Unfortunately, detection using interactions between molecules is often not specific and similar molecules can interfere with the analyte of interest. Therefore, two detection spots (cytochrome and TiO_2_) were placed on the surface of the chip. The cytochrome showed a greater sensitivity to glyphosate than to AMPA. In the contrary, detection on TiO_2_ had the same sensitivity for both the monitored substances. Dividing the signal from both the measuring spots, therefore, made it possible to determine whether only glyphosate was present in the mixture or whether its degradation product AMPA was present (Figure 5).

The standard solutions of glyphosate at different concentrations (between 10–100 mg L^−1^) showed a cytochrome/TiO_2_ SPR signal ratio of 3.0, whereas AMPA had a cytochrome/TiO_2_ ratio close to 1.0. At the beginning of the photolytic and photocatalytic experiments, the cytochrome/TiO_2_ ratio was close to 3.0 for both treatments. At the end of the experiments, the photocatalytic samples showed a significantly lower value of 1.5, whereas the photocatalytic samples had their cytochrome/TiO_2_ ratio only slightly reduced to 2.6. This suggested that photocatalysis produced more AMPA than photolysis.

HPLC only measures the concentration of known compounds, for which analytical standards are available, while TOC measures the concentration of all possible intermediates. The final concentration of glyphosate was the lowest, as determined by HPLC–MS, in both the photolytic and photocatalytic experiments. The values obtained by detection using the SPRi sensor were higher than the glyphosate concentrations measured by HPLC but lower than the values obtained by the TOC method. This corresponded to the principle of the methods, where the HPLC method must be targeted at known compounds, while TOC analysis measures all the carbon present in the sample. SPRi sensing connects both approaches, i.e., it not only targets specific compounds using a suitable ligand bound to the biochip surface but also gives information about other nonidentified molecules present in the sample if the detection spot is also located outside the ligand, and the response is given by the refractive index of the measured sample.

By using specific molecules or nanoparticles to enhance SPR, it is also possible to reduce the detection limit of the sensor [41,42,43]. For glyphosate detection, several highly sensitive sensors based on the SPR phenomenon have been proposed recently, e.g., a localized surface plasmon resonance (LSPR) sensor using anisotropic gold nanoparticles with gold nanobipyramids [44], a phase-sensitive SPR sensor with internal referencing [45], or an electrochemical surface plasmon resonance (ESPR) sensor based on a molecularly imprinted polymer deposition on a gold chip/electrode [46]. All these sensors aim for improved sensitivity and good selectivity towards glyphosate. However, the SPRi method theoretically allows the placement of tens to hundreds of spots [47,48] with different compounds on the surface of the biosensor, which makes it possible to achieve a better targeting of the analysis to specific compounds. Therefore, this approach may be suitable for the detection of molecules with low molecular weights during the research of the photolytic and photocatalytic degradation of emerging pollutants in model solutions, where the detection limit in the order of milligrams per liter is usually sufficient.

## 4. Conclusions

In our study, we developed an effective SPRi method for monitoring the process of the photolytic and photocatalytic degradation of glyphosate dissolved in water, which could be applied in a flow-through arrangement, and thus detect changes taking place in real-time. We proved that SPRi sensing was a suitable method for these applications and could be widely used in the study of xenobiotic removal from water. The advantages of using the SPRi method include the speed of determination (in the order of minutes), the possibility of miniaturization of the equipment and thus its portability, and last but not least its low financial costs and demands in terms of operator skills compared to chromatographic methods. We believe that the proposed SPRi method could be widely used in the study of xenobiotic removal from surface water or wastewater.

## Data Availability

The data presented in this study are available on request from the corresponding author.

## Supplementary Materials

The following supporting information can be downloaded at: https://www.mdpi.com/article/10.3390/s22239217/s1, Figure S1: Calibration curves of SPR signal (reflectivity) for glyphosate (a,b) and AMPA (c,d) detected on cytochrome (a,c) and TiO_2_ spots (b,d); Figure S2: Ratio of SPR signal on cytochrome and TiO_2_ for model samples containing a mixture of glyphosate and AMPA, expressed as a percentage of the glyphosate content in the sample. The total concentration of glyphosate and AMPA in the samples was 100 mg L^−1^.
