# Supplementary material for: Surface Plasmon Resonance Imaging Sensor for Detection of Photolytically and Photocatalytically Degraded Glyphosate
Source: Sensors (Basel). 2022 Nov 27;22(23):9217. doi: 10.3390/s22239217 (PMC9738441; doi:10.3390/s22239217)
Supplement: Supplementary file 1 [file sensors-22-09217-s001.zip › sensors-2043199-supplementary.pdf]

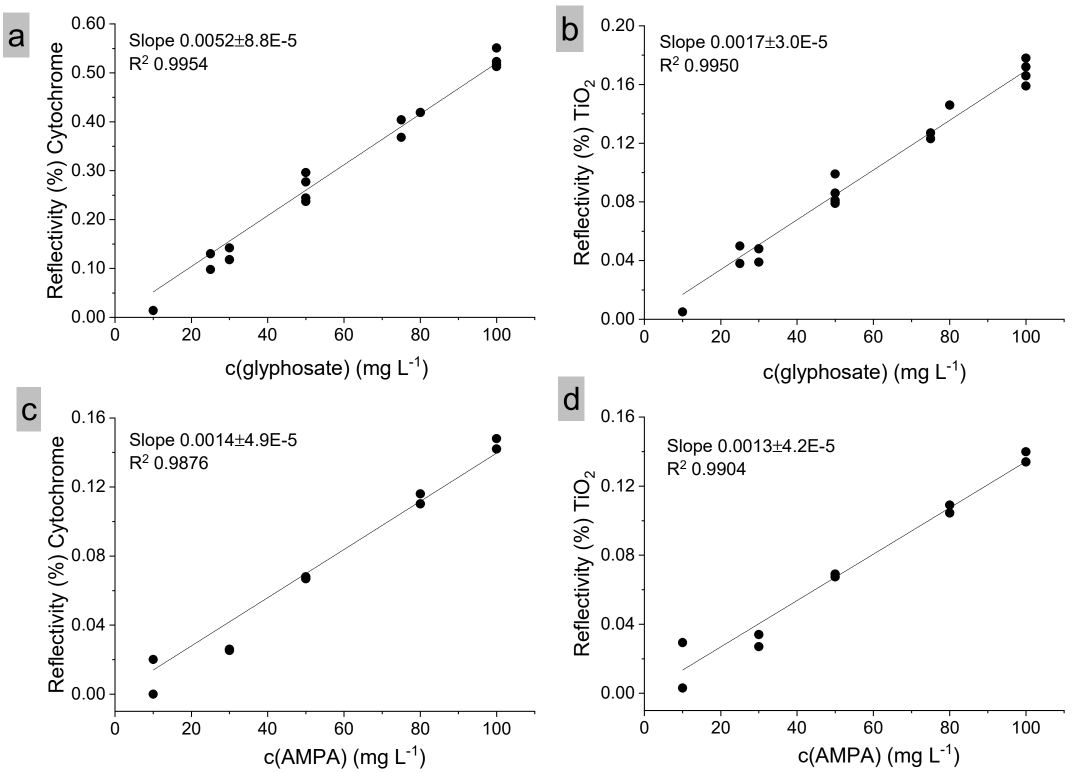

Figure S1. Calibration curves of SPR signal (reflectivity) for glyphosate (a,b) and AMPA (c,d) detected on cytochrome (a,c) and TiO_2 spots (b,d).

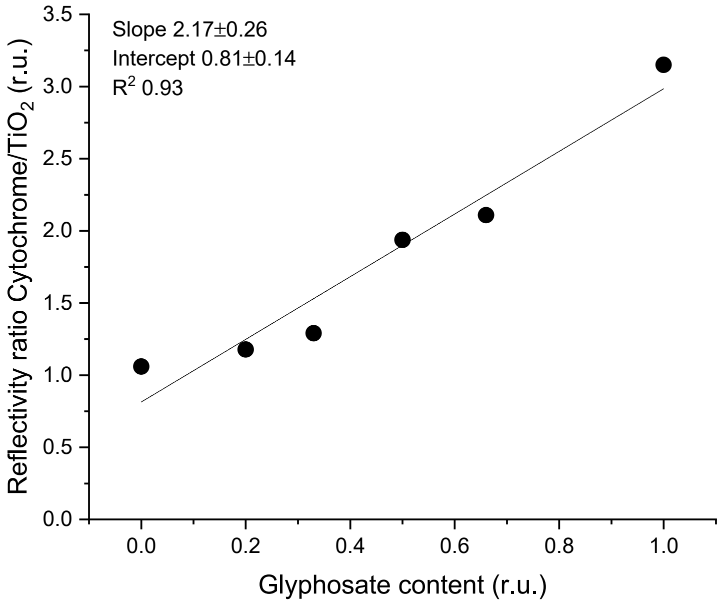

Figure S2. Ratio of SPR signal on cytochrome and TiO_2 for model samples containing a mixture of glyphosate and AMPA, expressed as a percentage of the glyphosate content in the sample. The total concentration of glyphosate and AMPA in the samples was 100 mg L^{-1}.
